# Phosphatidylethanolamine Protects Nucleus Pulposus Cells From Oxidative Stress‐Induced Cellular Senescence and Extracellular Matrix Degradation by Promoting Autophagy

**DOI:** 10.1002/jsp2.70058

**Published:** 2025-04-10

**Authors:** Yijun Dong, Chuanfu Li, Shuangshuang Tu, Mingkai Liu, Kai Lv, Liqun Duan, Feng Zhang, Haiping Cai, Xi Chen, Wenzhi Zhang

**Affiliations:** ^1^ Department of Orthopedics Provincial Hospital Affiliated to Anhui Medical University, Anhui Medical University Hefei China; ^2^ Clinical College of Anhui Medical University of China Hefei China; ^3^ College of Pharmacy Anhui Xinhua University Hefei China; ^4^ Department of Laboratory Medicine, The First Affiliated Hospital Shihezi University Shihezi Xinjiang China; ^5^ Department of Orthopedics, First Affiliated Hospital, School of Life Sciences Bengbu Medical University Bengbu China; ^6^ Department of Orthopedics, The First Affiliated Hospital of USTC, Division of Life Sciences and Medicine University of Science and Technology of China Hefei China

**Keywords:** autophagy, cellular senescence, intervertebral disc degeneration, metabolomics analysis, phosphatidylethanolamine

## Abstract

**Background:**

Intervertebral disc degeneration (IDD) is a type of musculoskeletal system diseases that prevail widely in human society, exerting a substantial economic burden on society. The extensive aggregation of senescent nucleus pulposus (NP) cells within the discs is a significant characteristic of lumbar degenerative alterations. Exploring the underlying mechanisms of NP cell senescence and developing strategies to retard cell senescence are anticipated to become effective approaches for the treatment of IDD.

**Objective:**

The study aims to investigate the effects of phosphatidylethanolamine (PE) on autophagic activity, cellular senescence, as well as IDD and dedicated to forging an evidence chain that interconnects IDD, the senescence of NP cells, the autophagic malfunction of NP cells, and the aberrant PE content in NP cells of the advanced‐stage group. The resultant outcomes will furnish a theoretical underpinning for the biological prophylaxis and treatment of IDD.

**Methods:**

Oxidative stress‐induced NP cells senescence is a fundamental characteristic of IDD. To obtain a understanding of the metabolite profile changes in NP cells under stress conditions, Liquid Chromatograph/Mass Spectrometer‐based untargeted metabolomics (LC/MS) analysis was utilized in this study. Upon analysis, the distinctive metabolite, PE, which decreased in content in advanced‐stage cells, was identified. In this study, Tert‐Butyl hydroperoxide (TBHP) was selected as the oxidant to construct an in vitro cellular oxidation model. Methods such as immunofluorescence, immunohistochemistry, Western blotting, and transmission electron microscopy were employed to explore the effects of PE on the senescence of NP cells, the degradation of the extracellular matrix (ECM), and the autophagy of NP cells under stress conditions.

**Results:**

The administration of PE effectively attenuates TBHP‐induced cellular senescence and ECM degradation in NP tissue, primarily by stimulating autophagy. Nonetheless, this restorative effect is hindered by chloroquine (CQ), a lysosomal alkalizing agent.

**Conclusions:**

In our study, a series of experiments established a conclusive evidential chain linking IDD, senescence of NP cells, impaired cellular autophagy activity, and abnormal PE content within advanced‐stage NP cells. The unique function of PE in promoting NP cells autophagy, thereby delaying cellular senescence, restoring cellular homeostasis, and ECM, suggests its potential as an effective drug for the clinical treatment of IDD.

## Graphical abstract

In this study, we utilized LC/MS‐based untargeted metabolomics analysis to examine changes in the metabolite profiles of NP cells under stress conditions. Upon analysis, PE was identified as a differentially abundant metabolite for its decreased content within advanced‐stage NP cells. A series of experiments have demonstrated the capacity of PE to enhance the autophagic activity of NP cells under stress conditions, thereby inhibiting cell senescence and ECM degradation.

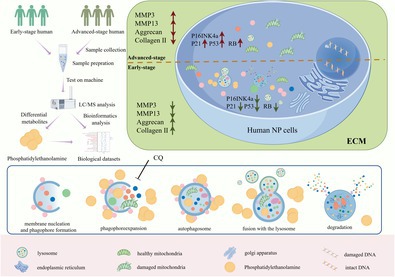

## 1 Introduction

Intervertebral disc degeneration (IDD) is a common and enduring musculoskeletal disease that imposes a substantial economic burden on society [1, 2]; however, a limited understanding of its pathogenesis has hindered the efficacy of therapeutic interventions. Previous research has indicated that the accumulation of senescent nucleus pulposus (NP) cells in discs is a distinguishing feature of IDD [3]. The accumulation of senescent cells in the disc disrupts the intricate balance between anabolic and catabolic processes within the extracellular matrix (ECM), ultimately leading to the collapse of the intervertebral space and spinal dysfunctions. However, the definitive molecular mechanisms underlying cellular senescence remain poorly understood. Hence, elucidating the intricate mechanisms underlying cellular senescence and formulating strategies to postpone cellular senescence hold great potential as a therapeutic approach for IDD.

Autophagy has been demonstrated to be strongly correlated with NP cell senescence, as evidenced by the observation that the suppression of autophagic activity hastens the process of cellular senescence in human NP cells [4, 5, 6, 7]. Various pathological conditions, including abnormal mechanical stress, inflammatory cytokines, and metabolic disturbances, have the potential to compromise the physiological functions of NP cells, leading to the accumulation of impaired organelles or dysfunctional proteins in cells [8]. In the presence of these pathological stimuli, autophagy is triggered as a protective mechanism to maintain cellular energy equilibrium and eliminate damaged organelles or dysfunctional components. The initial step in the autophagy process involves the creation of a double‐membraned autophagosome, achieved through the elongation of an isolation membrane that encapsulates cellular contents [9]. The development of the autophagy membrane is a complex and highly dynamic process that can be influenced by various stressors, including fluctuations in the cellular microenvironment, organelle malfunctions, and the accumulation of reactive oxygen species (ROS). When these factors impede the formation of the autophagosome membrane, cells may enter a state of senescence [10]. However, little is known about the relationships among stress conditions, cellular senescence, and cellular autophagy activity.

Metabolomic analysis is a valuable method for discerning changes in metabolic profiles linked to stress conditions [11, 12]. To elucidate the metabolic profiling shifts in NP cells under conditions induced by oxidative stress, an untargeted metabolomics analysis utilizing LC/MS was conducted, revealing a reduction in phosphatidylethanolamine (PE) content in advanced‐stage NP cells. PE, which comprises approximately 15%–25% of the total phospholipids in cells, plays a crucial role in cell membrane structure and carries out various biological functions [13, 14, 15]. In the context of autophagy, PE serves as a key component of autophagosome/autolysosome membranes and plays a significant biological role in the autophagic process. The covalent attachment of PE to LC3, followed by its binding to the autophagosome, forms the basis for the cellular autophagic process [9, 13]. Thus, a series of experiments was undertaken to explore the associations among PE, cellular senescence, and autophagy in human NP cells.

## 2 Materials and Methods

### 2.1 NP Samples and Cell Culture

This study was conducted in accordance with the Declaration of Helsinki and the Orthopedic Research Society (ORS) Spine guidelines. Human NP samples were obtained from patients who underwent discectomy at the First Affiliated Hospital of the University of Science and Technology of China (USTC). The collection of all samples was approved by our institution's ethics committee (No. 2020‐KY‐19), and informed consent was secured from each patient. Detailed information regarding these patients is provided in Table S1.

The grade of each sample was assessed using an MRI‐based Pfirrmann grading system, with Grades I –II collectively categorized as early‐stage disease and Grades III –V classified as advanced‐stage disease. Prior to enzymatic digestion, the collected human NP tissue was sectioned into fragments and rinsed twice with PBS. Following a 4‐h incubation in 0.25% type II collagenase for enzymatic preparation, the cells were filtered through a 70 μm cell strainer and resuspended in DMEM/F12 medium. The DMEM/F12 medium was supplemented with 10% fetal bovine serum (FBS), 100 U/mL penicillin, and 100 mg/mL streptomycin, maintained at 37°C in a humidified atmosphere comprising 95% air and 5% CO_2_ [16]. The culture medium was replaced every 3 days, and the cells were passaged at passage two.

Regarding the formulation of the cellular model, the cells were cultured following the aforementioned protocol and tert‐butyl hydroperoxide (TBHP) was chosen as the peroxide to induce stress conditions. After counting, the NP cells were seeded at a density of 2 × 10^4^ cells per cm^2^ in 6‐well plates. When the cells recovered to a healthy state, 50 μM TBHP was introduced into 1 mL of DMEM/F12 medium and incubated with 2 × 10^5^ NP cells for a period of 2 h. The preparation of the PE solution was carried out as follows: the PE was re‐suspended in chloroform at a concentration of 20 mg/mL and stored at −80°C. Prior to use, the required amount of PE was evaporated under nitrogen gas, followed by the addition of an equivalent volume of DMEM/F12 medium containing 1% albumin to the resulting pellet, which was then sonicated at 4°C for 2 min. For the PE + chloroquine (CQ) solution, given that CQ is soluble in DMEM/F12 medium, the requisite amount of CQ was directly incorporated into the DMEM/F12 medium to maintain equivalent concentrations of PE and CQ. Subsequently, the PE + CQ solution was subjected to sonication prior to its utilization in the study.

### 2.2 MDC Staining

The autophagy of NP cells was detected by an autophagy staining assay kit with MDC (Beyotime). According to the manufacturer's protocol, the MDC staining solution was incubated with the cells for 30 min at 37°C in a light‐free environment. After being washed three times with assay buffer, the cells were examined using a confocal microscope.

### 2.3 EdU Staining

NP cell proliferation was assessed via a BeyoClick EdU‐488 staining kit (Beyotime). Briefly, the NP cells were incubated with EdU working solution at 37°C for 12 h [17]. After the samples were fixed in 4% paraformaldehyde and permeabilized with 0.3% Triton X‐100, the newly prepared click reaction solution was added to the confocal cultures and incubated with the cells for 30 min in the dark. Subsequently, Hoechst 33342 was used to stain the nuclei of the cells. The degree of cell proliferation is presented as the percentage of positively stained cells relative to the total number of cells in each field of the slides.

### 2.4 Senescence‐Associated β‐Galactosidase (SA‐β‐Gal) Staining

NP cell senescence was determined by an SA‐β‐Gal staining kit (Beyotime). According to the manufacturer's protocol, the cells grown in 6‐well plates were fixed with SA‐β‐Gal fixation solution at room temperature for 15 min and then incubated with SA‐β‐Gal staining solution at 37°C without a CO_2_ supply overnight.

### 2.5 Lysosomal Staining

Lysosomes were labeled with LysoTracker (Beyotime). Briefly, 50 nM LysoTracker was added to DMEM/F12 and incubated with NP cells at 37°C for 30 min [18]. The NP cells were rinsed twice with DMEM/F12 solution and then immediately examined for fluorescence expression in viable cells.

### 2.6 CCK‐8 Viability Assay

Cell viability was assessed by using a CCK‐8 viability kit (Beyotime). The NP cells were cultured in 96‐well plates at a density of 5 × 10^3^ cells per well for 48 h, followed by treatment with drugs under the specified conditions. The reaction was performed in a dark environment for 2 h, and optical density (OD) values were detected at 450 nm.

### 2.7 ROS

The intracellular level of ROS was determined using an ROS assay kit (Beyotime). In accordance with the manufacturer's protocol, DCFH‐DA was diluted in serum‐free medium and incubated with the cells at 37°C for 20 min.

### 2.8 Cell Cycle Assay

The cell cycle distribution of the NP cells was assessed via a cell cycle assay (Abbkine). Briefly, the NP cell masses were washed with precooled PBS, centrifuged at 600 × g for 5 min, resuspended in precooled 70% ethanol, and stored at −20°C overnight. After centrifugation and resuspension, the cells were suspended in staining solution and incubated in the dark for 30 min prior to detection.

### 2.9 Transmission Electron Microscopy (TEM)

For TEM analysis, the sample preparation was set as previously described [17]. Specifically, the NP samples were fixed in 2.5% glutaraldehyde overnight, treated with 1% osmium tetroxide at 4°C for 2 h, and then dehydrated with a series of graded ethanol solutions (from 30%, 50%, 70%, 90% to 100% ethanol). After dehydration, the samples were treated with embedding medium that contained propylene oxide. After ultrathin sectioning by using an LKB‐V ultramicrotome and staining with uranyl acetate and lead citrate, the samples were positioned onto a transmission electron microscope for observation.

### 2.10 Western Blotting (WB) Analysis

The cell pellets were solubilized in RIPA buffer, and the protein concentrations were determined by using a BCA protein assay kit (Beyotime). After separation by SDS–PAGE, the proteins were transferred to PVDF membranes. Following blocking and incubation with a specific primary antibody, the membranes were incubated with a horseradish peroxidase (HRP)‐conjugated secondary antibody. A chemiluminescence detection kit was used to visualize the images. The expression level of GAPDH was utilized for normalization to the relative expression levels of other proteins by using ImageJ software.

### 2.11 Metabolomic

Our metabolomic analysis was conducted by Lu‐Ming Biotech, with samples processed in accordance with the standard protocol (Shanghai). For the LC/MS analysis, raw data obtained from LC/MS were extracted and processed using Progenesis QI software (Waters Corporation, Milford, MA, USA). The following parameters were applied: precursor tolerance of 5 ppm, fragment tolerance of 10 ppm, and a production threshold of 5%. This resulting matrix was utilized to construct three‐dimensional datasets encompassing retention time, mass‐to‐charge ratio (*m*/*z*), and normalized ion intensities. The matrix underwent further reduction by eliminating any peaks with missing values (ion intensity = 0) present in more than 50% of the samples. Metabolites were identified through Progenesis QI Data Processing Software based on public databases such as http://www.hmdb.ca/; http://www.lipidmaps.org/, along with self‐constructed databases from Ouyi Corporation (Shanghai, China). Both positive and negative data sets were amalgamated and imported into R's ropls package for analysis. Principal component analysis (PCA) and orthogonal partial least squares‐discriminant analysis (OPLS‐DA) were performed to visualize alterations between groups. Differential metabolites were selected from the OPLS‐DA model utilizing a combination of VIP value > 1 and *p* < 0.05 determined via two‐tailed Student's t‐test on normalized peak areas.

### 2.12 Animals

In this study, all animal experiments were approved by the committee of the First Affiliated Hospital of USTC (No. 2024‐N(A)‐169), and the animals were handled in accordance with the methods of the Laboratory Animal Anesthesia and the guidelines set forth by the American Veterinary Medical Association. A total of 20 healthy male Sprague–Dawley rats (SD rats, 200 ± 20 g, 8 week old) were purchased from the Animal Center of USTC, and the rats were fed regularly for 2 months. Prior to surgical intervention, we meticulously monitored the rats' daily behavioral patterns, including their eating and drinking habits, weight fluctuations, and tail vertebrae thickness.

The SD rats were randomly allocated into 4 groups of 5 rats each, namely, the Control group, IDD group, IDD + PE group, and IDD + PE + CQ group. Following isoflurane anesthesia, a rat model was established by puncturing the intervertebral disc at the Co6 –Co7 level using a 20‐gauge needle. The needle was inserted vertically into the rat's tail, traversed through the contralateral skin, and then rotated 360°for a duration of 30 s [18, 19, 20, 21]. Subsequently, the needle was withdrawn. The depth of penetration was meticulously controlled at 5 mm to ensure that the tip of the needle reached the NP region. The IDD + PE group received an intradiscal injection of a 1 μL solution of PE into the intervertebral disc. The IDD + PE + CQ group was administered a 1 μL solution of PE and CQ via intradiscal injection. Normal saline was injected into the discs of the IDD group. All drug injections were performed once weekly for a total duration of 4 weeks using a microinjector with a volume capacity of 1 μL, an outer tip diameter of 0.7 mm, and a needle length of 75 mm. The postoperative activity and wound healing of the experimental rats were meticulously monitored over time, with no complications arising during the procedures. The rats involved in the experiment were euthanized via carbon dioxide inhalation, and their carcasses were disposed of in a manner that ensured environmental safety.

### 2.13 Radiographic Evaluation

Both preoperative and postoperative measurements of internal control discs were conducted concurrently with their corresponding punctured discs. Specifically, disc height and the heights of adjacent vertebral bodies were measured at the midline and 25% of the disc's width from the midline on each side. The Disc Height Index (DHI) was calculated as the mean of three measurements taken from the midline to the boundary of the central 50% of disc width, divided by the mean height of two adjacent vertebral bodies. Changes in DHI for punctured discs were expressed as a percentage (%DHI = post‐punctured DHI/pre‐punctured DHI × 100) [18].

For MRI analysis, sagittal T_2_‐weighted images in the midsagittal plane of the rats were acquired to evaluate the severity of disc degeneration. The MRI was conducted using a 3.0 T system (GE, USA) to obtain T_2_‐weighted images with parameters set at a repetition time of 2500 ms, echo time of 158 ms, field of view measuring 320 × 320 mm, and slice thickness of 0.8 mm. The degree of degeneration changes was evaluated according to the Pfirrmann grading system [22].

### 2.14 Histological Staining and Immunostaining Assays

Human NP tissue samples were fixed in paraformaldehyde for 24 h, decalcified using an EDTA solution for 1 month, dehydrated, and subsequently embedded in paraffin. The human NP tissue samples were sectioned into 5 μm thick slices and subjected to hematoxylin–eosin (HE) and Masson staining following their respective standard protocols. For the animal model, specific rat coccygeal discs were harvested through analogous procedures. Rat coccygeal disc sections were stained with HE staining, Masson stain, and Alcian blue staining.

Immunohistochemical (IHC) staining of 5‐μm‐thick slices from humans or animals was performed as described previously. Endogenous enzymes and antibodies in tissues were blocked by using IHC blocking solution to minimize background staining and reduce false‐positive staining. Specific antibodies were used to bind to the target protein at 4°C overnight, after which the sample sections were incubated with secondary antibodies at room temperature for 1 h and then re‐dyed with hematoxylin. The images were magnified by a factor of 50, and random image acquisition was conducted using a double‐blind methodology.

For IF staining, the NP cells were fixed with 4% paraformaldehyde and permeabilized using 0.25% Triton X‐100 at room temperature. After being blocked indoors with 5% BSA, the NP cells were incubated with a specific antibody at 4°C overnight. Subsequently, the NP cells were treated with Alexa Fluor 488 or Alexa Fluor 594 secondary antibodies. The cell nuclei were counterstained with DAPI solution prior to the acquisition of representative images.

### 2.15 Histological Score

In this study, histological scoring was conducted in accordance with the recommendations of the ORS Spine [23]. Briefly, the human intervertebral disc was histologically evaluated across four parts: NP, annulus fibrosus (AF), cartilage endplate, and bone endplate. Each part received a score ranging from 0 to 9 based on criteria including cellularity characteristics (0–3), lesion presence (0–3), and ECM structure (0–3). The scores for each intervertebral disc part were independently performed by three experienced team members, followed by the calculation of the score of two members with similar evaluations to establish a degeneration grade, resulting in three classifications: non‐degenerate (0–3), mid‐grade degeneration (4–6), and severe degeneration (7–9) [23].

In the evaluation of intervertebral disc degeneration in rats, a novel grading system was implemented, which incorporated histological assessment of the disc across five distinct parameters: NP morphology (0–4), NP cellularity (0–4), NP –AF border integrity (0–2), AF morphology (0–4), and endplate condition (0–2) [24].

### 2.16 Statistical Analysis

Apart from the cell cycle analysis, the data in this article represent the mean ± SD of at least three independent biological replicates. The data from the cell cycle analysis are presented as the means. Statistical analysis was performed using GraphPad 9.0 software. Data from two groups with normal or nonparametric distribution were subjected to Student's two‐tailed *t*‐test or Mann–Whitney nonparametric test, respectively. For multi‐group comparisons, ANOVA was applied for normally distributed data or the Kruskal –Wallis test for non‐normally distributed data. To eliminate potential subjective biases, this experiment was conducted and analyzed using a double‐blind methodology. *p* < 0.05 was considered to indicate a statistically significant difference.

## 3 Results

### 3.1 Autophagic Dysfunction Was Detected in IDD Patients

**FIGURE 1:**
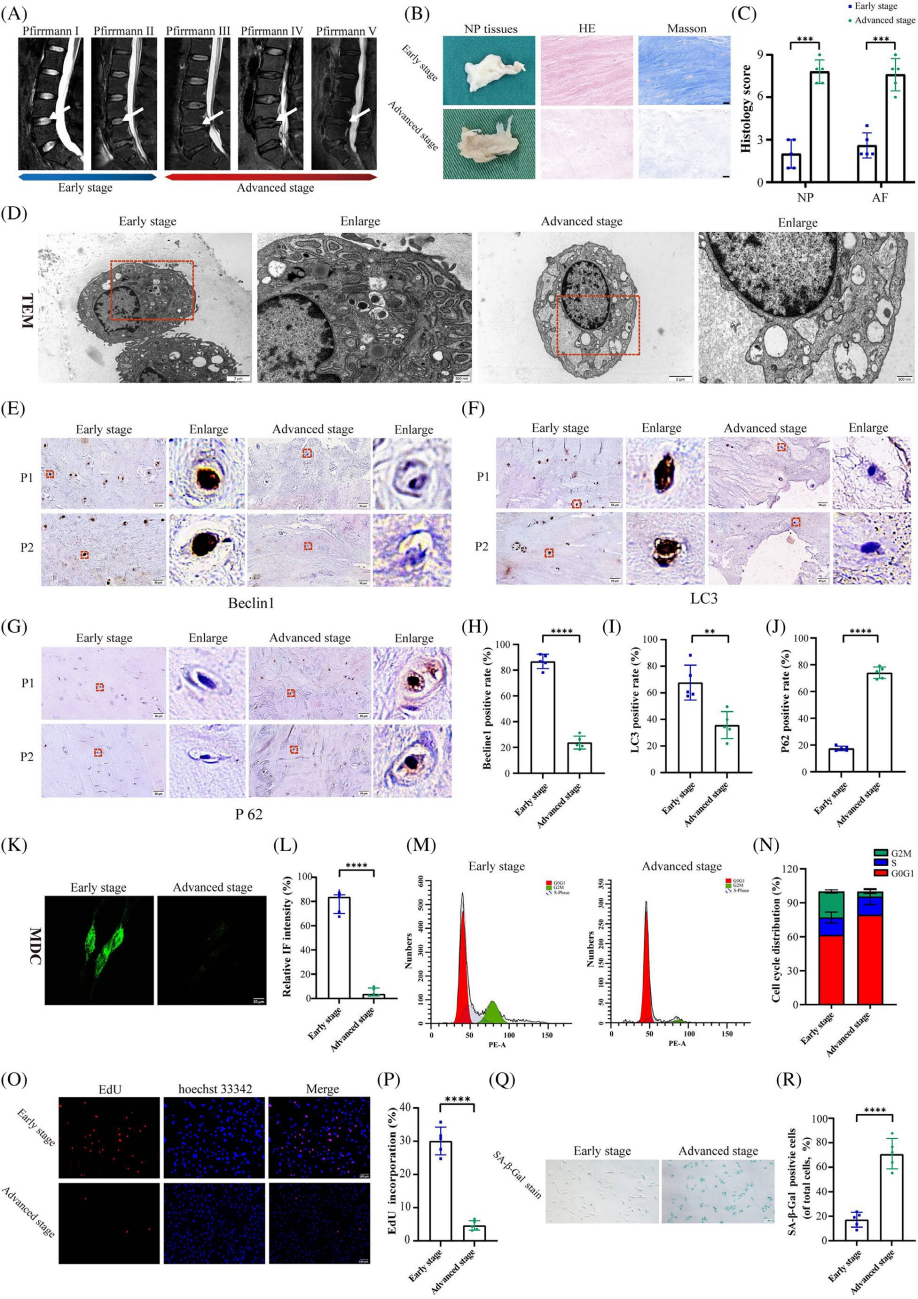
Autophagic dysfunction was detected in IDD patients. (a) Representative human T_2_‐weighted images from grades I to V. The degenerative discs are indicated by white arrows. (b, c) The histological changes in early‐ and advanced‐stage NP tissues were measured by HE and Masson staining. Scale bar: 50 μm. *n* = 5 independent biological replicates. ***p < 0.001. (d) TEM images revealed cellular structural damage and autophagy dysfunction within advanced‐stage NP cells. Scale bars: 2 μm and 500 nm. *n* = 5 independent biological replicates. (e–j) IHC results of Beclin1, LC3 and p62 as well as MDC staining revealed impaired autophagy activity in advanced‐stage NP cells. Scale bars: 50 μm. *n* = 5 independent biological replicates. ****p < 0.0001. (m, n) Analysis of cell cycle phases and quantitative analysis of samples from the two stage groups. *n* = 3 independent biological replicates. (o–r) The senescence status of the advanced‐stage group cells was validated through EdU and SA‐β‐Gal staining. Scale bars: 100 and 200 μm. *n* = 5 independent biological replicates. ****p < 0.0001.

The severity of disc degeneration changes was demonstrated in T_2_‐weighted images. Compared to those of advanced‐stage discs, early‐stage discs exhibit greater hydration and normal intervertebral space (Figure 1a). Representative histological characteristics of NP tissues through HE and Masson staining are shown in Figure 1b. The cells in the early‐stage group exhibited a stellar morphology enveloped by dense ECM, whereas those in the advanced‐stage group presented a small and flat shape with sparse ECM (Figure 1b,c). In addition, a TEM analysis was used to observe the microstructure of the NP cells and assess their cellular states. In contrast to those in the early‐stage group, the cells in the advanced‐stage group exhibited severe structural damage (Figure 1d). It can be observed that the cells in the early‐stage group possess intact cell membranes and abundant organelles. The abundant organelles included not only mitochondria, endoplasmic reticulum, and Golgi apparatus, but also contained numerous autophagosomes and/or lysosomes, suggesting that autophagy is occurring within the early‐stage group cells. Conversely, the NP cells in the advanced‐stage group exhibit pronounced tortuosity and swelling of the membrane structure, alongside a notable loss of intracellular organelles. The impaired physiological condition of the cell is correlated with autophagy dysfunction in cells. Furthermore, an assay of several autophagic marker proteins, including Beclin1, LC3, and p62, was conducted. Both Beclin1 and LC3 were weakly positive, while p62 levels were increased, indicating the impaired autophagy function in advanced‐stage cells (Figures 1e–j and S1). The lack of MDC staining within advanced‐stage cells also confirmed the results (Figure 1k–l). In addition, multiple senescence phenotypes, such as cell cycle arrest, a low percentage of EdU‐positive cells, and an increase in the percentage of SA‐β‐Gal‐positive cells, were all found in advanced‐stage cells, collectively providing evidence supporting their senescence status (Figure 1m–r). All of these findings consistently demonstrate impaired autophagic activity in advanced‐stage NP cells, underscoring the correlation between NP cell senescence and impaired autophagic activity in IDD.

### 3.2 Metabolomic Analysis Revealed the Changes in Metabolite Profiles Among Patients With IDD

**FIGURE 2:**
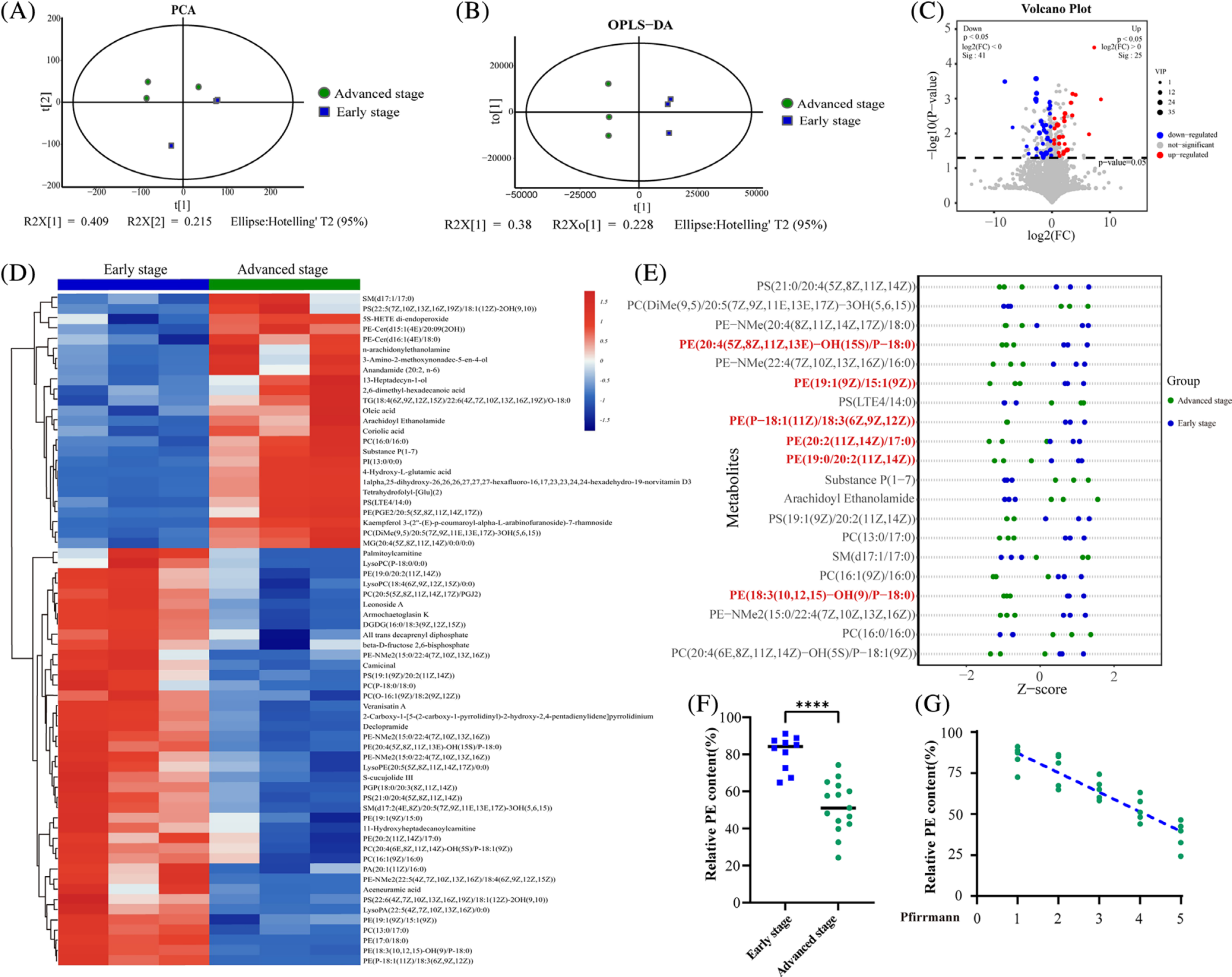
Metabolomic analysis revealed the changes of metabolite profiles among patients with IDD. (a, b) The PCA and OPLS‐DA analysis of the two stage group samples. *n* = 3 independent biological replicates. (c, d) The volcano and heat map analysis of differential metabolites between the two stage groups were conducted with a threshold of VIP > 1 and *p* < 0.05. (e) Z score analysis of the top 20 diverse abundant metabolites, as ranked by VIP. The species of PE are highlighted in red font. (f) Analysis of the relative PE content between the two stage group samples. (g) The PE contents were inversely correlated with the Pfirrmann grades in human NP samples. *n* = 5 independent biological replicates. *y* = −0.1192 × *x* + 0.9914, R2 = 0.8324, ****p < 0.0001.

In our study, LC/MS‐based untargeted metabolomics analysis was utilized to investigate the changes in metabolite profiles in IDD [11, 12, 17, 25]. PCA and OPLS‐DA were performed to visualize metabolite profile alterations between groups(Figure 2a,b). Differential metabolites were identified from the OPLS‐DA model by applying a combination of VIP > 1 and *p* < 0.05, determined through a two‐tailed Student's *t*‐test on normalized peak areas. Upon analysis, as illustrated by the volcano map and the heat map, a total of 66 metabolites exhibited variance in content between groups(Figure 2c,d). In addition, a Z‐score analysis of the top 20 metabolites was conducted, as ranked by VIP, and the results demonstrated that the abundance of PE in advanced‐stage cells was lower than that in the early‐stage cells (Figure 2e). To further validate the findings, an additional cohort of patients was selected for analysis of the relative PE content and there was a notable disparity in abundance within the PE, which gradually decreased as the Pfirrmann grade increased [17] (Figure 2f,g).

### 3.3 PE Protects NP Cells From Cellular Senescence and Mitigates ECM Degradation

**FIGURE 3:**
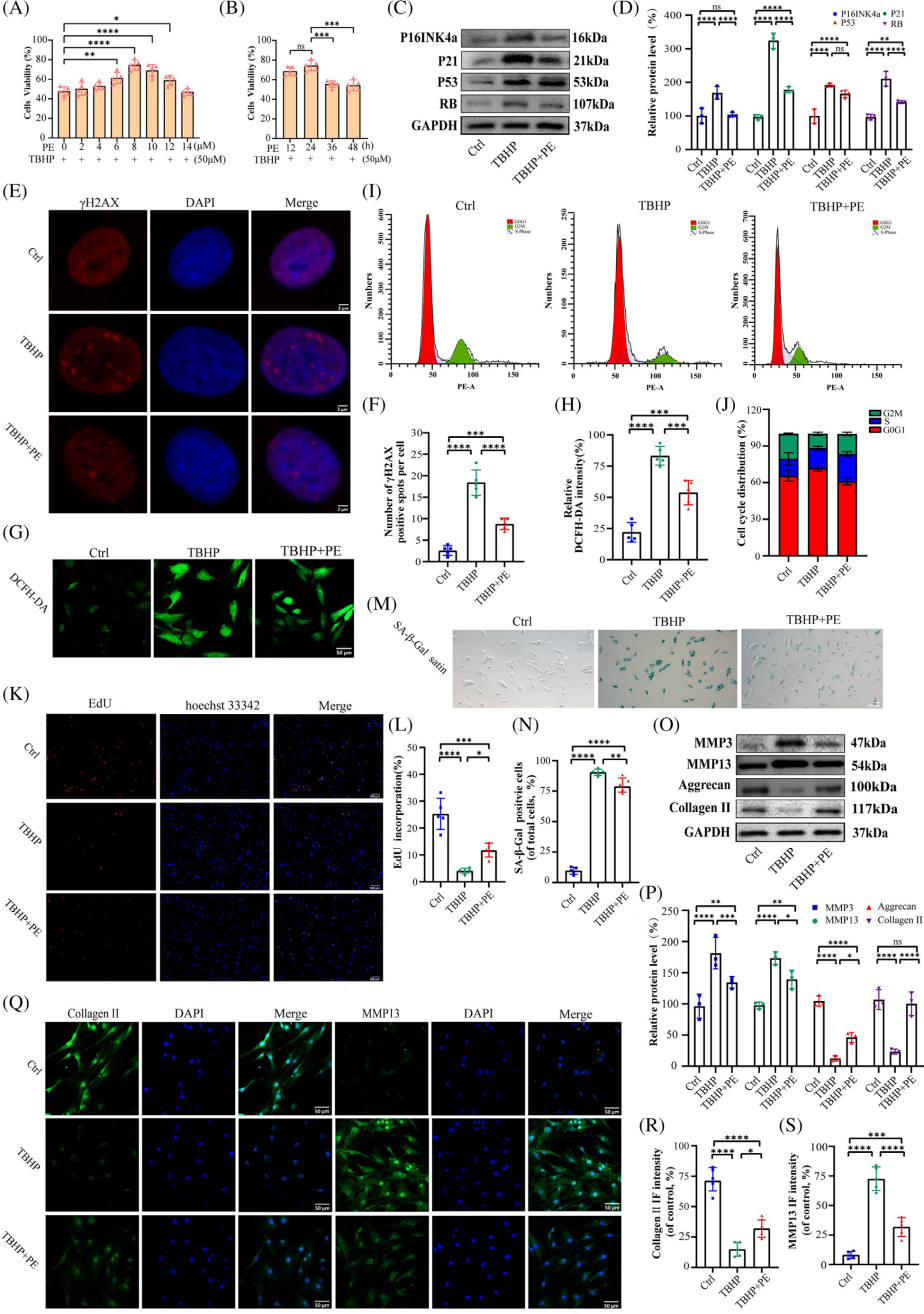
PE protects NP cells from cellular senescence and mitigates ECM degradation. (a) The CCK‐8 assay results for NP cells subjected to treatment with PE and TBHP over a 24‐h period. *n* = 5 independent biological replicates. *p < 0.05, **p < 0.01, ****p < 0.0001. (b) The CCK‐8 assay results for NP cells treated with 8 μmol of PE and TBHP over a period of 12 to 48 h. *n* = 5 independent biological replicates. ***p < 0.001. (c, d) The WB results and quantitative analysis of p16INK4a, p21, p53, RB and GAPDH with the indicated treatments. *n* = 3 independent biological replicates. **p < 0.01, ****p < 0.0001. (e–n) Representative images of γH2AX, ROS, SA‐β‐Gal and EdU with the indicated treatments. *n* = 5 independent biological replicates. Scale bars: 2, 50, 200 and 100 μm. *p < 0.05, **p < 0.01, ***p < 0.001, ****p < 0.0001. (o, p) The WB results and quantitative analysis of collagen II, aggrecan, MMP3, MMP13 and GAPDH with the indicated treatments. *n* = 3 independent biological replicates. *p < 0.05, **p < 0.01, ***p < 0.001, ****p < 0.0001. (q–s) Representative images of collagen II and MMP13 with the indicated treatments. *n* = 5 independent biological replicates. Scale bar: 50 μm *p < 0.05, **p < 0.01, ***p < 0.001, ****p < 0.0001.

Although studies have suggested that PE plays an indispensable role in the human body [13, 14, 15], its involvement in IDD remains to be understood. In this study, TBHP was utilized to establish an in vitro model [18, 26]. As indicated by the CCK‐8 assay, there was an initial upward trend in cell viability, followed by a gradual decline. Notably, when the PE content was 8 μmol and incubated for 24 h, the cells exhibited optimal growth. Thus, it was chosen as the suitable condition for subsequent experiments (Figure 3a,b). In addition, PE and albumin were respectively added to the NP cells without TBHP supply to evaluate their effects on NP cells. The findings indicated that a low dose of PE (≤ 2 μmol) facilitated the proliferation of NP cells, whereas a high dose (> 2 μmol) produced an opposing effect. Albumin did not exhibit an impact on the states of NP cells (Figure S2a,b). Consistent with senescent NP cells in vivo, TBHP‐stimulated NP cells also exhibit multiple senescence characteristics, including cell cycle arrest, macromolecular damage, and upregulation of the senescence‐associated secretory phenotype (SASP) [25, 27, 28, 29]. The results of the WB analysis indicated that post‐PE treatment, despite the lack of statistical significance in P53, the relevant indicators of cellular senescence, including p16INK4a, p21, and RB, all exhibited a declining trend (Figure 3c,d). IF analysis of γH2AX and DCFH‐DA indicated that the treatment of PE attenuated TBHP‐induced DNA damage and decreased intracellular ROS levels (Figure 3e–h). In addition, cell cycle analysis revealed an increase in the proportion of PE‐treated cells in the G2/M and S phases of the cell cycle [27, 28, 29] (Figure 3i–j). The results of the EdU and SA‐β‐Gal assays further support the aforementioned findings (Figure 3k–n). In terms of ECM degradation, WB and IF analyses both showed that TBHP stimulation reduced the protein expression of collagen II and aggrecan; in contrast, it promoted the expression of matrix metalloproteinases (MMP3 and MMP13), but PE partially reversed these effects (Figure 3o–s). In conclusion, PE has a beneficial effect on delaying NP cellular senescence and restoring the balance between ECM synthesis‐related proteins and degradation‐related proteins to alleviate ECM degradation.

### 3.4 PE Promotes Autophagy in NP Cells Under Stress Conditions

**FIGURE 4:**
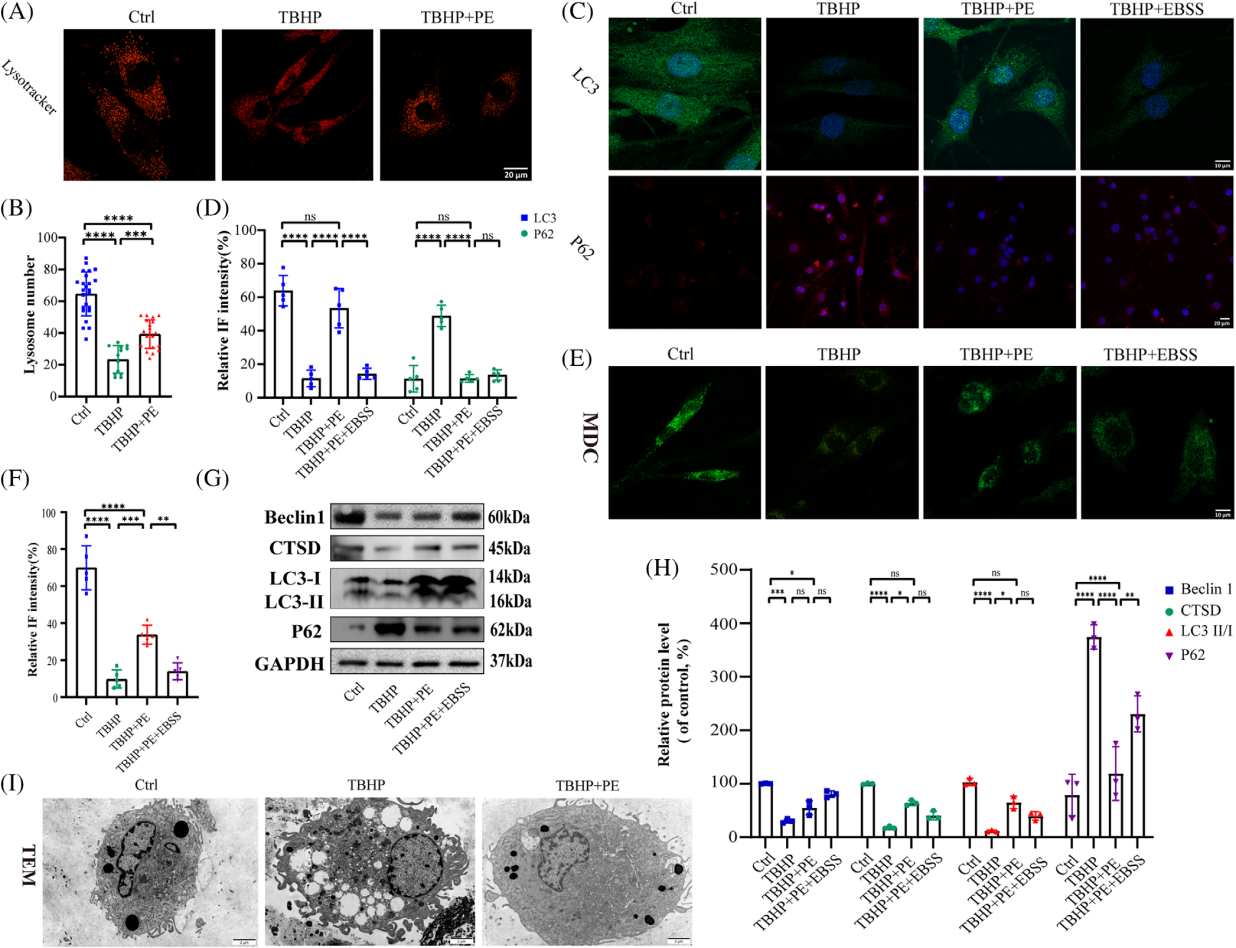
PE promotes autophagy in NP cells under stress conditions. (a, b) Representative images and quantification of LysoTracker Red in NP cells with the indicated treatments. Scale bar: 20 μm. ***p < 0.001, ****p < 0.0001. (c, d) Representative images of LC3 and p62 in NP cells with the indicated treatments. Scale bars: 10 and 20 μm. *n* = 5 independent biological replicates. ***p < 0.001, ****p < 0.0001. (e, f) MDC staining of the cells with the indicated treatments. Scale bars: 10 μm. *n* = 5 independent biological replicates.**p < 0.01, ***p < 0.001, ****p < 0.0001. (g, h) The WB results and quantitative analysis of Beclin1, CTSD, LC3, P62 and GAPDH in cells with the indicated treatments. *n* = 3 independent biological replicates. *p < 0.05, **p < 0.01, ***p < 0.001, ****p < 0.0001. (i) Representative TEM images of the samples with the indicated treatments. *n* = 5 independent biological replicates. Scale bar: 2 μm.

Promoting the autophagy activity of NP cells is anticipated to be an effective therapeutic intervention for IDD. As indicated by LysoTracker staining, there was a decreased number of lysosomes in TBHP‐stimulated cells, which was increased by PE treatment (Figure 4a,b). Based on previous research, Earle's balanced salt solution (EBSS) was selected as an autophagy inducer [30, 31]. Under TBHP stimulation, the IF analysis of LC3 and P62 together revealed the compromised autophagic activity in NP cells. Specifically, the impaired autophagic activity was partially restored by treatment with PE (Figure 4c,d). Additionally, with TBHP‐induced stimulation, reduced LC3 fluorescence intensity within the nucleus was observed in our study; it was also restored through PE treatment, but this effect was not detected in EBSS‐treated cells. Consistent findings were confirmed by MDC staining, namely, PE treatment partially restored autophagy dysfunction in TBHP‐induced NP cells (Figure 4e,f). Several autophagy‐related proteins, including autophagosome markers (Beclin1 and LC3), the lysosomal enzyme cathepsin D (CTSD), and an autophagic substrate protein (p62), were analyzed (Figure 4g,h). WB analysis revealed an increase in the LC3‐II/I protein ratio and CTSD expression in PE‐treated cells, but p62 expression decreased. This finding suggests that, in comparison to EBSS, PE has beneficial effects on lysosomal hydrolases and a pronounced impact on the induction of autophagy in NP cells. No statistical difference in Beclin1 levels was observed between the cells treated with PE and those stimulated with TBHP. However, a noticeable increase was observed in the EBSS‐treated cells. This indicated that, in contrast to EBSS, the PE‐induced autophagy in NP cells is not associated with decreased mTORC1 activity or AMPK activation [13, 32, 33]. TEM analysis further revealed an increased cellular state within PE‐treated NP cells (Figure 4i). All of these results consistently showed that PE promoted autophagy in NP cells subjected to TBHP stimulation.

### 3.5 PE Alleviated NP Cellular Senescence and Mitigated ECM Degradation via the Lysosome Pathway

**FIGURE 5:**
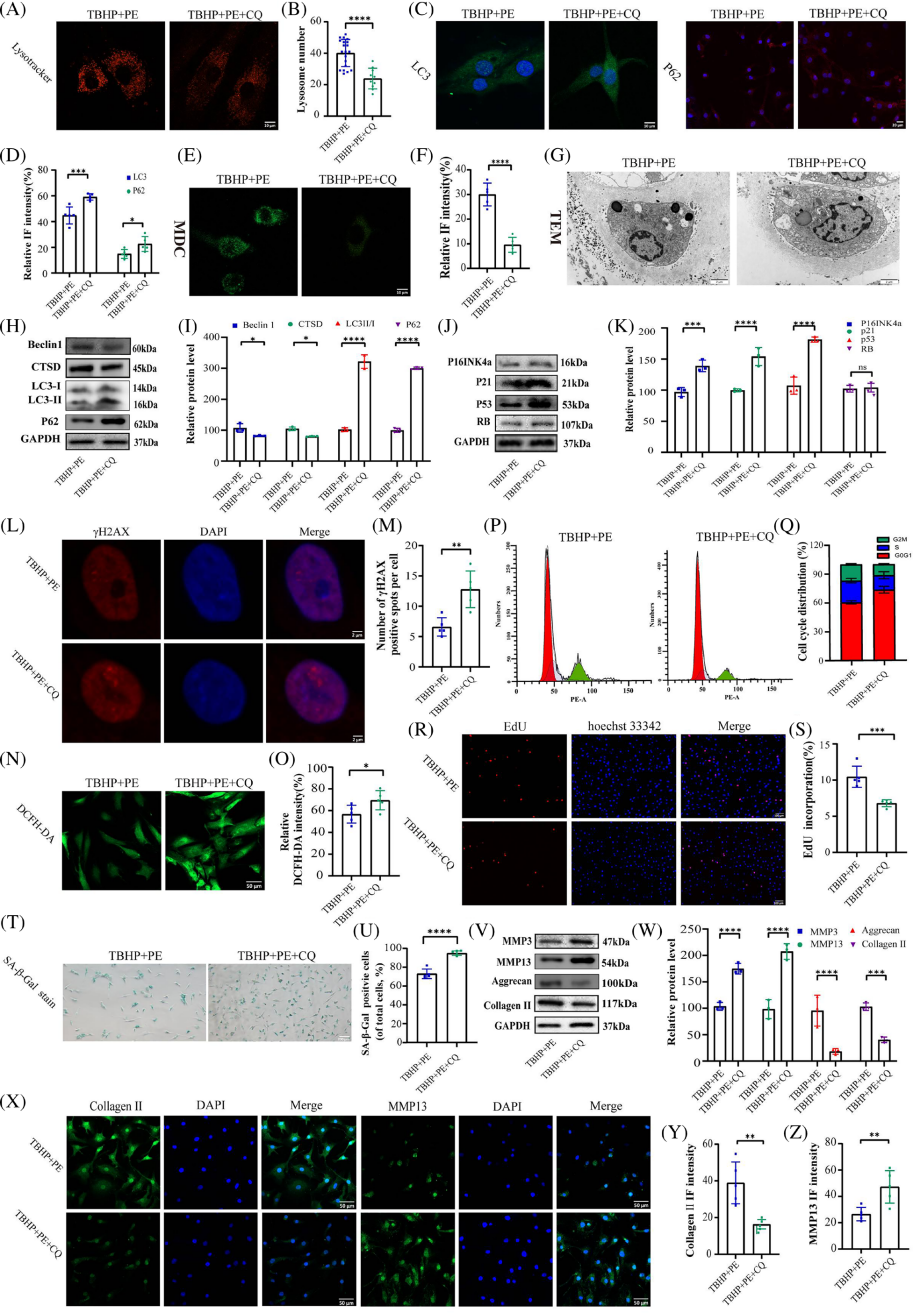
PE alleviated NP cellular senescence and mitigated ECM degradation via the lysosome pathway. (a, b) Representative images and quantification of LysoTracker Red in NP cells with the indicated treatments. Scale bars: 10 μm. ****p < 0.0001. (c–f) Representative images of LC3, p62, and MDC staining of samples with the indicated treatments. *n* = 5 independent biological replicates. Scale bars: 10, 20 and 10 μm. *p < 0.05, ***p < 0.001, ****p < 0.0001. (g) Representative TEM images of the samples with the indicated treatments. *n* = 5 independent biological replicates. Scale bars: 2 μm and 500 nm. (h, i) The WB results and quantitative analysis of Beclin1, CTSD, LC3, P62 and GAPDH with the indicated treatments. *n* = 3 independent biological replicates. *p < 0.05, ****p < 0.0001. (j, k) The WB results and quantitative analysis of p16INK4a, p21, p53, RB and GAPDH with the indicated treatments. *n* = 3. ***p < 0.001, ****p < 0.0001. (l–o) γH2AX and ROS staining of cells with the indicated treatments. *n* = 5 independent biological replicates. Scale bars: 2 and 50 μm. *p < 0.05, **p < 0.01. (p, q) Analysis of cell cycle phases with the specified treatment. *n* = 3 independent biological replicates. (r–u) EdU and SA‐β‐Gal staining of cells with the indicated treatments. *n* = 5 independent biological replicates. Scale bars: 100 and 200 μm. ***p < 0.001, ****p < 0.0001. (v, w) The WB results and quantitative analysis of collagen II, aggrecan, MMP3, MMP13 and GAPDH with the specified treatment. *n* = 3 independent biological replicates.***p < 0.001, ****p < 0.0001. (x–z) Representative images of collagen II and MMP13 with the specified treatment. Scale bar: 50 μm. *n* = 5 independent biological replicates. **p < 0.01.

To investigate the potential benefits of PE‐induced autophagy on delaying cellular senescence and ECM degradation, we examined the impact of coculturing PE with CQ, a lysosomal alkalizing agent [26]. As an autophagy inhibitor, CQ elevates the pH of lysosomes and inactivates acid hydrolases within these organelles, thereby impeding the fusion and degradation of autophagolysosomes in cells. As shown in Figure 5a,b, the application of CQ has resulted in a reduction in the number of lysosomes and inhibited autophagy in NP cells. The increased fluorescence intensity of LC3 and P62, along with the absence of MDC staining, collectively provided evidence for the compromised autophagic activity in CQ‐treated cells (Figure 5c–f). Similar findings were also noted in the WB assays (Figure 5h–j). TEM analysis further revealed that the presence of CQ disrupted the microstructure of the NP cells (Figure 5g).

As for the effects of PE on delayed cellular senescence and ECM degradation, it was also inhibited by CQ. It is embodied in the upregulation of cellular senescence markers in CQ‐treated cells, including p16INK4a, p21, and p53, increased DNA damage, the accumulation of ROS, and even cell cycle arrest (Figure 5j–q). Consistently, EdU and SA‐β‐Gal staining confirmed these results. For the ECM, CQ counteracted the protective effect of PE, leading to a disruption in the delicate balance between anabolic and catabolic processes (Figure 5r–u). Decreased levels of collagen II and aggrecan, along with increased expression of MMP3 and MMP13, were demonstrated by both IF and WB assays (Figure 5v–z). These findings suggest that PE enhances its beneficial effects by promoting the autophagic activity of NP cells under stress conditions, a process that is impeded by the autophagy inhibitor CQ.

### 3.6 PE Inhibited Disc Degeneration In Vivo

**FIGURE 6:**
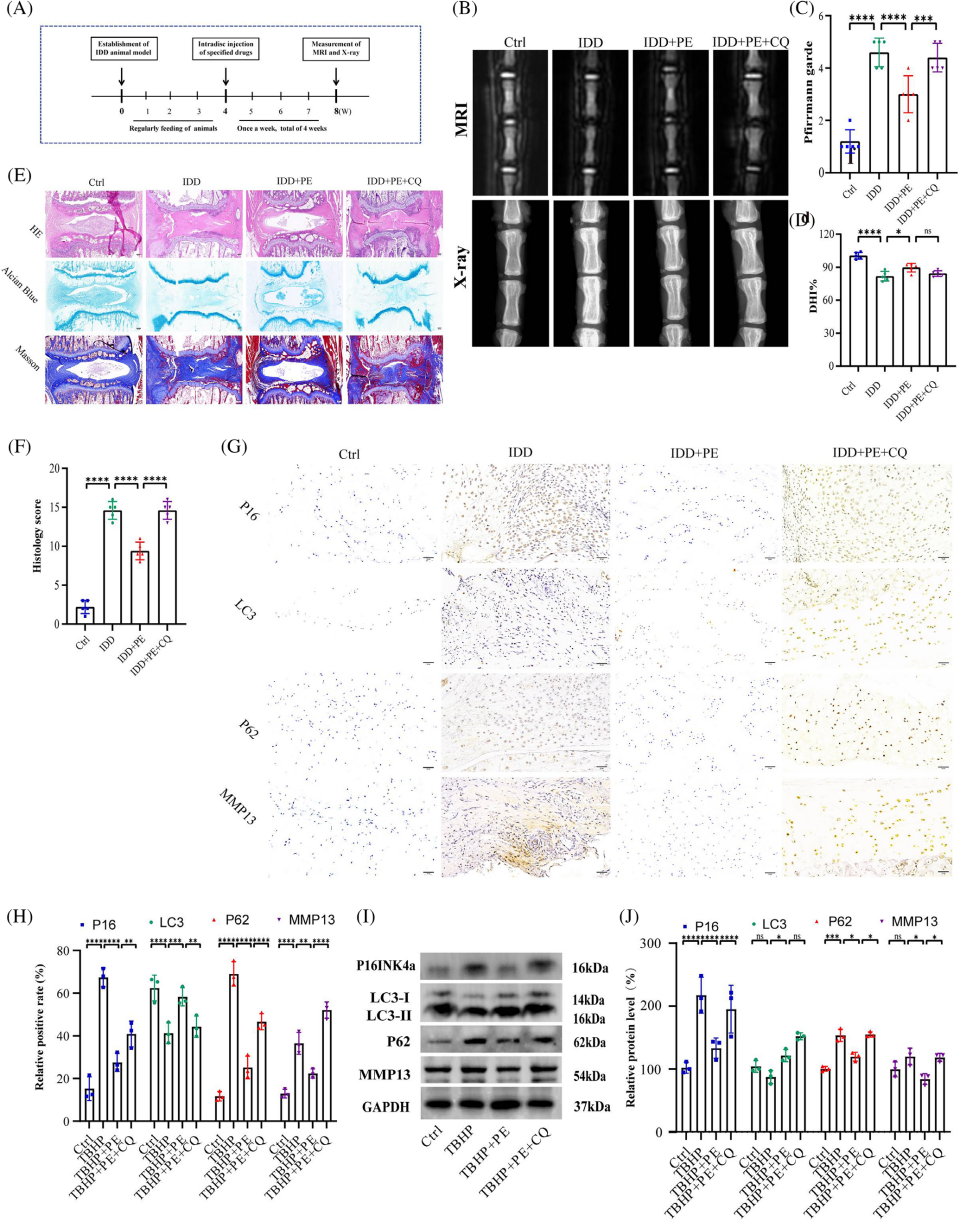
PE inhibited disc degeneration in vivo. (a) Schematic illustration of the animal experimental design. (b) Representative X‐ray and MR T2‐weighted images of the coccygeal vertebrae in rats subjected to the specified treatment. (c, d) Pfirrmann grading and DHI% were utilized to assess the degree of degeneration in the coccygeal vertebrae. *n* = 5 independent biological replicates. *p < 0.05, ***p < 0.001, ****p < 0.0001. (e, f) HE, Masson and Alcian blue staining were performed on the coccygeal vertebrae of rats subjected to the indicated treatments, and the histological score was determined. *n* = 5 independent biological replicates. ****p < 0.0001. (g, h) IHC staining of p16, LC3, p62, and MMP13 in coccygeal discs from rats subjected to the indicated treatments. *n* = 3 independent biological replicates. **p < 0.01, ***p < 0.001, ****p < 0.0001. (i–j) The WB results of LC3 and p62 supported the occurrence of autophagy in the IDD + PE group. The downregulation of P16 and MMP13 highlights the impact of PE in delaying cellular senescence and facilitating the recovery of the ECM. *n* = 3 independent biological replicates. *p < 0.05, ****p < 0.0001.

To further assess the potential function of PE in vivo, a needle puncture‐induced rat model was established. The procedures were performed as previously described, and a schematic illustration is shown in Figure 6a. Radiological analysis, including MRI and X‐ray, was conducted to evaluate degenerative changes. Compared with those in the IDD group, increased T2‐weighted intensity and DHI% were found in the IDD + PE group. However, this effect was partly counteracted by CQ (Figure 6b–d). The grading system of Lai et al. was used to evaluate histological changes [24]. As determined by HE, Masson, and Alcian blue staining, the discs in the IDD group exhibited a reduction in NP area, disorganization of the AF, and a relatively higher degenerative histological grade. Interestingly, the rats treated with PE exhibited an abundance of NP cells, reduced fragmentation, and pronounced staining of the ECM. As similar with the radiological results, this effect was also hindered by CQ (Figure 6e,f). Subsequently, IHC analysis of P16, LC3, P62, and MMP13 was conducted for further assessment. The decrease in the percentage of P62‐positive cells and increase in the percentage of LC3‐positive NP cells supported the occurrence of active autophagy in the IDD + PE group. The downregulation of P16 and MMP13 highlights the positive impact of PE in delaying cellular senescence and facilitating the recovery of the ECM. Similarly, these phenomena are disturbed by CQ (Figure 6g,h). The identical outcome was corroborated by WB analysis(Figure 6i–j). Collectively, these findings demonstrate that PE exerts its beneficial effects through the promotion of autophagy. The accumulation of PE in intervertebral discs shows promise as a potential therapeutic strategy for the treatment of IDD.

## 4 Discussion

Various stress conditions have been identified as predisposing factors for the impairment of autophagy, which triggers a cascade of pathogenic responses that disrupt the normal function of NP cells and ultimately lead to IDD. Among these stress conditions, the induction of cellular senescence through ROS is collectively considered a hallmark of degenerative diseases [3, 34]. In‐depth exploration of the regulatory mechanism of autophagy under stress conditions is anticipated to delay cellular senescence and provide insights for IDD treatment. Simultaneously, due to the structural integrity provided by the AF and cartilaginous endplates, the NP tissue resides in a relatively enclosed environment in vivo. The avascular nature of NP tissue exposes the cells to a hypoxic, acidic, and nutrient‐deprived microenvironment [35]. Consequently, the metabolic profiles of NP cells undergo significant alterations that render them susceptible to entering a senescent state. However, comprehensive information on overall metabolic profile changes during NP cell senescence remains scarce, hindering further insights into the role of autophagy in IDD. Therefore, an analysis encompassing global metabolic profile changes during IDD progression may yield overall data reflecting the impact of stress conditions on cellular homeostasis while elucidating intricate regulatory relationships between stress conditions and NP cell senescence.

Metabolomics is an emerging area of research within the omics field that focuses on the investigation of metabolic pathways in biological systems. Its biological functions include not only the analysis of endogenous metabolite metabolic pathways within organisms, organs, or tissues but also the assessment of metabolic profile changes induced by internal and external stimuli [11, 17]. By analyzing the changes in the overall metabolic profile, a series of biological processes occurring in specific pathophysiological pathways were identified. Comprehensive analysis of metabolic profile changes and these specific pathophysiological pathways is regarded as an effective way to treat diseases. The metabolome, in contrast to other omics studies, is positioned downstream of the gene regulatory network and the protein interaction network. It directly reflects biochemical activity and provides an accurate representation of an organism's terminal phenotypic information. Thus, we conducted an LC/MS‐based untargeted metabolomics analysis to explore changes in the metabolite profiles of early‐ and advanced‐stage NP cells to identify a new method for treating IDD.

Through this analysis, a distinct metabolite, PE, was identified for its decreased content within advanced‐stage NP cells. PE, as the second most abundant phospholipid in mammalian membranes, along with other bioactive lipids, contributes to cell membrane structural integrity and biological functions [13, 14, 15, 36]. However, the alteration of the cellular membrane composition results in an impairment of its biological functionality and initiates a series of subsequent reactions. For instance, changes in phospholipid composition in tumor cells lead to an increase in negative charges on the outside of the tumor cell membrane, promoting mutual repulsion and facilitating detachment [37, 38]. Similarly, the elevated levels of phospholipids and cholesterol in tumor cells result in changes in membrane fluidity and permeability, making it challenging for chemotherapy drugs to penetrate the cells [38]. Nevertheless, the physiological alterations in NP cells induced by PE reduction are still not fully understood.

Previous research has demonstrated various advantages in the context of PE. For instance, in A549 lung cancer tumor cells, inhibiting the synthesis of PE from phosphatidylserine was shown to impede tumor growth [39, 40]. Additionally, Rockenfeller et al. noted that inhibiting the expression of either of the two yeast phosphatidylserine decarboxylases effectively reduced intracellular PE levels and accelerated chronological aging‐associated ROS production and death. Elevating PE levels in cells by providing the precursor ethanolamine or overexpressing the PE‐producing enzyme phosphatidylserine decarboxylase 1 was also proposed to extend the lifespan of mammalian cells [13]. At the same time, it is essential to recognize the diverse roles of PE in physiological responses. Research on lipid‐induced endoplasmic reticulum (ER) stress and ferroptosis has revealed a delicate balance between saturated and unsaturated lipids within ER membranes [41]. An excess of PE species with polyunsaturated acyl chains in the ER membrane triggers the formation of toxic PE hydroperoxides that lead to cell death. Despite the extensive research conducted on PE thus far, our understanding of its impact on NP cells remains limited.

The biogenesis of autophagosomes commences with the formation of a cup‐shaped membrane vesicle and relies on the elongation of the autophagic membrane. Recent research using yeast as the main model organism proposed and verified that acetyl‐CoA synthetases play a crucial role in synthesizing phospholipids necessary for autophagy [13]. This finding indicates that the cells are relatively deficient in phospholipids and lays the groundwork for supplementing NP cells with exogenous phospholipids to enhance autophagosome membrane formation. In addition, a range of proteins has been implicated in the regulation of cellular autophagy, and most of these proteins are conserved in mammals. One of these components, LC3, is covalently attached to the headgroup of PE, anchoring it to the developing autophagosomal membrane. The lipidation of LC3 affects the membrane dynamics of autophagosomes and plays a significant role in determining their size. However, the impact of PE on the autophagic activity of NP cells remains poorly understood.

The lysosome, as a distinctive organelle within cells, is characterized by a singular membrane housing a variety of nonspecific hydrolases. Under the impact of various pathogenic factors, changes in the permeability of lysosomal membranes occur, resulting in hydrolase leakage and cellular structural damage [34]. Moreover, the completion of autophagy heavily relies on the fusion of autophagosomes with lysosomes, and the status of lysosomes has a great influence on this process. According to previous research, an increased Pfirrmann grade often correlates with a decreased number of lysosomes, indicating impaired autophagy in NP cells [34, 42, 43]. Although the repair of damaged lysosomal hydrolysis and restoration of enzyme activity are areas worthy of further exploration, the relevant information remains limited. Hence, we investigated the impact of exogenous PE supplementation on the lysosomal status of NP cells under stress conditions.

To validate our metabolomics analysis findings and the distinct physiological functions of PE in regulating autophagy activity, we selected TBHP as the ROS donor to induce stress conditions and used PE in human NP cells to investigate its association with cellular senescence. Diverse observations, including reduced SA‐β‐gal activity, DNA damage, accumulation of ROS, and downregulation of senescence‐related proteins, such as p16INK4a, p21, and RB, collectively demonstrate the capacity of PE to delay senescence and restore cellular homeostasis. In addition, cell proliferation ability and the cell cycle were also restored. Regarding ECM regulation, PE has been shown to reverse the TBHP‐induced imbalance between ECM synthesis/degradation by promoting the expression of collagen II and aggrecan while inhibiting the expression of matrix degradation enzymes. Further analysis revealed that PE exerts these effects by enhancing autophagy activity in NP cells; however, this effect can be suppressed by CQ. Similar results were confirmed in animal models through in vivo experiments.

Even though our research findings are interesting, it is crucial to acknowledge and address several limitations. First, the sample size utilized for metabonomics analysis was relatively small, which means that the obtained metabonomic profile may only provide a partial representation of diverse metabolic changes. Second, in our experimental findings, we observed a decrease in NP cell viability beyond a specific threshold of PE content and coculture duration. The underlying mechanism behind this phenomenon remains unclear; however, we hypothesize that there might be a potential association with lipotoxicity [44, 45]. Further investigation is warranted to elucidate this relevant mechanism. Additionally, the frequent repetition of injections complicates the maintenance of consistent acupuncture points, consequently resulting in a certain degree of damage to the AF. Simultaneously, it is important to acknowledge that the destruction of intervertebral disc tissue is an intrinsic limitation associated with exogenous injection therapies; therefore, a smaller syringe was selected by our team to minimize injury. Combined with literature and experimental results, the influence of repeated injections is limited, and the experimental results are valid [46]. The intradiscal drug injection is an invasive procedure that restricts its clinical applicability. Approaches aimed at enhancing the endogenous PE content in discs require further investigation.

## 5 Conclusions

In our study, a series of experiments established a conclusive evidential chain linking IDD, senescence of NP cells, impaired cellular autophagy activity, and abnormal PE content within advanced‐stage NP cells. The unique function of PE in promoting NP cell autophagy, thereby delaying cellular senescence, restoring cellular homeostasis, and restoring the ECM, suggests its potential as an effective drug for the clinical treatment of IDD. Moreover, this study provides a foundation for further exploration of the pathological mechanisms underlying lumbar degenerative diseases and lays the groundwork for the biological prevention and treatment of IDD.

## Author Contributions

Conceptualization, X.C. and W.Z.; sample collection and processing, Y.D., C.L., and S.T.; development of methodology, Y.D., C.L., and M.L.; animal experiment, K.L. and L.D.; analysis of data, S.T., F.Z., and H.C.; writing, review, and/or revision of the manuscript: Y.D.

## Conflicts of Interest

The authors declare no conflicts of interest.

## Supporting information


**Figure S1.** The WB results of Beclin1, LC3 and p62 on the two stage group samples. (a) The WB results of Beclin1, LC3 and p62 revealed impaired autophagy activity in advanced‐stage NP cells. *p < 0.05, **p < 0.01, ****p < 0.0001.

**Figure S2.** Effect of PE and albumin on NP cell viability without TBHP supplementation. (a) The CCK‐8 assay results for NP cells subjected to treatment with PE over a 24‐h period. *n* = 5 independent biological replicates. **p < 0.01, ***p < 0.001, ****p < 0.0001. (b) The CCK‐8 assay results for NP cells treated with albumin over a 24‐h period. *n* = 5 independent biological replicates.

**Table S1.** Patient demographics.
**Table S2**. List of reagents and resources.

## Data Availability

The data are available upon reasonable request.
