# Supplementary figures and images for: Phosphatidylethanolamine Protects Nucleus Pulposus Cells From Oxidative Stress‐Induced Cellular Senescence and Extracellular Matrix Degradation by Promoting Autophagy
Source: JOR Spine. 2025 Apr 10;8(2):e70058. doi: 10.1002/jsp2.70058 (PMC12043014; doi:10.1002/jsp2.70058)

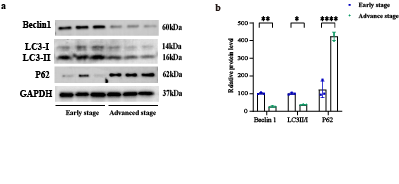

Supplement: Supplementary file 1 — Figure S1. The WB results of Beclin1, LC3 and p62 on the two stage group samples. (a) The WB results of Beclin1, LC3 and p62 revealed impaired autophagy activity in advanced‐stage NP cells. *p < 0.05, **p < 0.01, ****p < 0.0001. [file JSP2-8-e70058-s003.png]

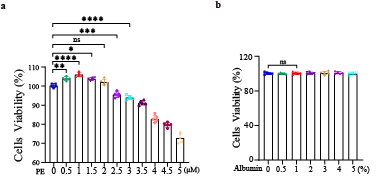

Supplement: Supplementary file 2 — Figure S2. Effect of PE and albumin on NP cell viability without TBHP supplementation. (a) The CCK‐8 assay results for NP cells subjected to treatment with PE over a 24‐h period. n = 5 independent biological replicates. **p < 0.01, ***p < 0.001, ****p < 0.0001. (b) The CCK‐8 assay results for NP cells treated with albumin over a 24‐h period. n = 5 independent biological replicates. [file JSP2-8-e70058-s002.png]
