# Supplementary material for: Phosphatidylethanolamine Protects Nucleus Pulposus Cells From Oxidative Stress‐Induced Cellular Senescence and Extracellular Matrix Degradation by Promoting Autophagy
Source: JOR Spine. 2025 Apr 10;8(2):e70058. doi: 10.1002/jsp2.70058 (PMC12043014; doi:10.1002/jsp2.70058)
Supplement: Supplementary file 3 — Table S1. Patient demographics. Table S2. List of reagents and resources. [file JSP2-8-e70058-s001.docx]

Table S1. Patient demographics

| Case | Gender | Age | Pfirrmann | Diagnosis | Figure |
| --- | --- | --- | --- | --- | --- |
| Case 1 | Male | 22 | I | Physical examination | Figure 1a |
| Case 2 | Male | 40 | Ⅱ | Backache | Figure 1a |
| Case 3 | Female | 44 | Ⅲ | Lumbar disc herniation | Figure 1a |
| Case 4 | Female | 77 | Ⅳ | Lumber degenerative disease | Figure 1a |
| Case 5 | Female | 79 | V | Lumber degenerative disease | Figure 1a |
| Case 6 | Female | 18 | Ⅱ | Lumbar disc herniation | Figure 1b,e,f,g |
| Case 7 | Male | 27 | I | Lumbar disc herniation | Figure 1b,e,f,g |
| Case 8 | Female | 30 | Ⅱ | Lumbar disc herniation | Figure 1b,e,f,g |
| Case 9 | Male | 16 | Ⅱ | Lumbar disc herniation | Figure 1b,e,f,g |
| Case 10 | Male | 20 | Ⅱ | Lumbar disc herniation | Figure 1b,e,f,g |
| Case 11 | Male | 66 | Ⅳ | Lumber degenerative disease | Figure 1b,e,f,g |
| Case 12 | Female | 70 | V | Lumber degenerative disease | Figure 1b,e,f,g |
| Case 13 | Male | 77 | V | Lumber degenerative disease | Figure 1b,e,f,g |
| Case 14 | Female | 69 | Ⅳ | Lumber degenerative disease | Figure 1b,e,f,g |
| Case 15 | Male | 59 | V | Lumber degenerative disease | Figure 1b,e,f,g |
| Case 17 | Female | 20 | Ⅱ | Lumbar disc herniation | Figure 2 |
| Case 18 | Female | 22 | Ⅱ | Lumbar disc herniation | Figure 2 |
| Case 19 | Male | 24 | Ⅱ | Lumbar disc herniation | Figure 2 |
| Case 20 | Female | 67 | V | Lumber degenerative disease | Figure 2 |
| Case 21 | Male | 76 | V | Lumber degenerative disease | Figure 2 |
| Case 22 | Male | 71 | V | Lumber degenerative disease | Figure 2 |
| Case 23 | Male | 27 | I | Lumbar disc herniation | Figure 2 |
| Case 24 | Male | 24 | I | Lumbar disc herniation | Figure 2 |
| Case 25 | Male | 16 | I | Lumbar disc herniation | Figure 2 |
| Case 26 | Male | 18 | I | Lumbar disc herniation | Figure 2 |
| Case 27 | Female | 21 | I | Lumbar disc herniation | Figure 2 |
| Case 28 | Male | 25 | Ⅱ | Lumbar disc herniation | Figure 2 |
| Case 29 | Female | 25 | Ⅱ | Lumbar disc herniation | Figure 2 |
| Case 30 | Male | 24 | Ⅱ | Lumbar disc herniation | Figure 2 |
| Case 31 | Male | 33 | Ⅱ | Lumbar disc herniation | Figure 2 |
| Case 32 | Male | 31 | Ⅱ | Lumbar disc herniation | Figure 2 |
| Case 33 | Male | 44 | Ⅲ | Lumbar spinal stenosis | Figure 2 |
| Case 34 | Female | 40 | Ⅲ | Lumbar disc herniation | Figure 2 |
| Case 35 | Male | 48 | Ⅲ | Lumbar disc herniation | Figure 2 |
| Case 36 | Female | 54 | Ⅲ | Lumbar spinal stenosis | Figure 2 |
| Case 37 | Female | 31 | Ⅲ | Lumbar disc herniation | Figure 2 |
| Case 38 | Male | 55 | Ⅳ | Lumbar spinal stenosis | Figure 2 |
| Case 39 | Female | 67 | Ⅳ | Lumbar disc herniation | Figure 2 |
| Case 40 | Female | 62 | Ⅳ | Lumbar spinal stenosis | Figure 2 |
| Case 41 | Male | 63 | Ⅳ | Lumbar disc herniation | Figure 2 |
| Case 42 | Female | 59 | Ⅳ | Lumbar disc herniation | Figure 2 |
| Case 43 | Male | 70 | V | Lumber degenerative disease | Figure 2 |
| Case 44 | Female | 73 | V | Lumber degenerative disease | Figure 2 |
| Case 45 | Male | 61 | V | Lumber degenerative disease | Figure 2 |
| Case 46 | Female | 69 | V | Lumber degenerative disease | Figure 2 |
| Case 47 | Male | 65 | V | Lumber degenerative disease | Figure 2 |
| Case 48 | Female | 33 | Ⅱ | Lumbar disc herniation | Cell culture and drug stimulation |
| Case 49 | Female | 29 | Ⅱ | Lumbar disc herniation | Cell culture and drug stimulation |
| Case 50 | Male | 31 | I | Lumbar disc herniation | Cell culture and drug stimulation |
| Case 51 | Male | 35 | Ⅱ | Lumbar disc herniation | Cell culture and drug stimulation |
| Case 52 | Male | 29 | Ⅱ | Lumbar disc herniation | Cell culture and drug stimulation |
| Case 53 | Female | 16 | Ⅱ | Lumbar disc herniation | Cell culture and drug stimulation |
| Case 54 | Female | 31 | Ⅱ | Lumbar disc herniation | Cell culture and drug stimulation |
| Case 55 | Female | 19 | I | Lumbar disc herniation | Cell culture and drug stimulation |
| Case 56 | Female | 30 | Ⅱ | Lumbar spinal stenosis | Cell culture and drug stimulation |
| Case 51 | Male | 25 | Ⅱ | Lumbar disc herniation | Cell culture and drug stimulation |
| Case 52 | Male | 29 | Ⅱ | Lumbar spinal stenosis | Cell culture and drug stimulation |
| Case 53 | Male | 31 | Ⅱ | Lumbar disc herniation | Cell culture and drug stimulation |

Table S2. List of reagents and resources.

| Reagents or Resources | Resource | Identifier |
| --- | --- | --- |
| Antibodies | | |
| anti-LC3 | HUABIO | JJ090-6 |
| anti-P62 | HUABIO | PS00-61 |
| anti-MMP13 | HUABIO | JF0893 |
| anti-Collagen II | HUABIO | ER1906-49 |
| anti-P16INK4a | Servicebio | GB111143-100 |
| anti-P21 | Servicebio | GB115313-100 |
| anti-P53 | Servicebio | GB12626-100 |
| anti-MMP3 | Servicebio | GB11131-100 |
| anti-γH2AX | Servicebio | GB111841-100 |
| anti-Aggrecan | Servicebio | GB11373-100 |
| anti-GAPDH | Servicebio | GB15004-100 |
| anti-Beclin1 | Beyotime | AF5123 |
| anti-CTSD | Proteintech | 21327-1-AP |
| anti-RB | Proteintech | 67521-1-Ig |
| Chemical | | |
| type II collagenase | Thermo Fisher | 17101015 |
| EBSS | Beyotime | C0213 |
| PE | MedChemExpress | HY-W250118 |
| CQ | MedChemExpress | HY-17589A |
| TBHP | MERYER | M25623 |
| Culture Medium | | |
| DMEM/F12 | Biosharp | BL305A |
| FBS | Absin | abs981 |
